# Topology-Controlled Dipolar Ordering and Wetting Thermodynamics of Water in Hydrophobic Nanotubes

**DOI:** 10.1021/acs.jpclett.6c01820

**Published:** 2026-07-22

**Authors:** Yuriy G. Bushuev, Mirosław Chorążewski

**Affiliations:** Institute of Chemistry, 431562 University of Silesia in Katowice, 40-006 Katowice, Poland

## Abstract

Classical capillarity does not fully describe water confined in hydrophobic nanopores, where nanoscale confinement fundamentally modifies the thermodynamics of the confined liquid. Using a family of model hydrophobic nanopores that enables independent control of wall thickness, confinement topology, and water–surface interaction strength, we demonstrate that water intrusion is governed not only by surface hydrophobicity but also by pore topology. Comparison of nanotubes with two open ends, shell-coated nanotubes, and nanotubes closed at one end separates the effects of the external shell and broken axial symmetry. The shell modifies the interaction environment surrounding the confined water, reducing the effective hydrophobicity of the nanopore, whereas closing one end stabilizes a single polarized water domain. Orientational ordering develops already in the liquid state, prior to crystallization, and contributes an additional electrostatic component to the free energy landscape. Consistent with the systematic reduction of intrusion pressure, the reconstructed free energy profiles indicate a progressive decrease in the estimated activation barrier from open to closed nanotubes. These results identify confinement topology as an independent parameter governing the thermodynamics of confined water and establish a direct connection between collective dipolar organization and intrusion behavior, providing new design principles for nanoporous and nanofluidic materials with tunable wetting properties.

## Graphical abstract

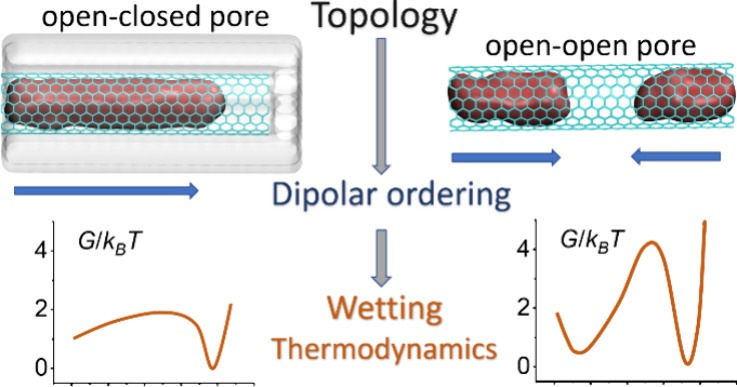

Fluids confined in nanoscale pores exhibit structural, dynamical, and thermodynamic properties that differ fundamentally from those of the bulk phase, reflecting the strong influence of confinement geometry and interfacial interactions. In particular, confinement can modify phase behavior, suppress fluctuations, and promote collective molecular organization, leading to distinct thermodynamic states of confined fluids.^1–3^

Water provides a particularly striking example due to its ability to form hydrogen-bonded networks and exhibit collective ordering under confinement.^4,5^ In nanoscale confinement, water displays pronounced deviations from bulk behavior, including structural reorganization, altered dynamics, and modified thermodynamic properties.^6–8^ In carbon nanotubes, such confinement leads to anomalously high mobility, including ultrafast and low-dissipation transport.^9–11^

The wetting of water in nanopores is typically described in terms of surface hydrophobicity and capillarity.^12–17^ However, at the nanoscale, this framework often becomes insufficient, as confinement-induced correlations and molecular ordering can substantially reshape the free energy landscape.^18–21^ In hydrophobic nanopores, wetting proceeds via pressure-driven intrusion rather than spontaneous adsorption from the vapor phase, leading to an intrusion–extrusion cycle instead of conventional adsorption–desorption behavior.^15,22^ Recent studies have further demonstrated that pore topology and connectivity can strongly influence intrusion–extrusion phenomena, particularly in hierarchical and interconnected nanoporous systems.^23–25^

At the same time, water under nanoscale confinement can exhibit pronounced dipolar ordering and collective orientational behavior, resulting in qualitatively different wetting and drying mechanisms.^5^ However, the interplay between molecular ordering and pore topology in determining the intrusion mechanism remains unclear. Here, we address this question by investigating water intrusion in model systems based on carbon nanotubes with controlled wall interactions, pore-wall thickness, and topology. We show that breaking axial symmetry by closing one end of the tube leads to a qualitative change in the intrusion–extrusion mechanism. This transition is associated with the emergence of collective dipolar ordering, which stabilizes the confined phase and modifies the thermodynamic pathway of intrusion.

The ability to convert confined fluid transformations into directed mechanical response is central to the development of nanoscale actuators and nanomotors.^26–28^ Most existing approaches rely on chemical reactions, interfacial gradients, or bubble-driven mechanisms. In contrast, the use of confined fluids as an energy storage and release medium remains largely unexplored, despite the potential of intrusion–extrusion cycles to generate mechanical work.^29^

These findings suggest that intrusion can be viewed as a topology-controlled transition between metastable confined states. Confinement topology, symmetry, and wall thickness therefore emerge as key parameters governing the behavior of confined water and provide a basis for controlling wetting, transport, and actuation in nanoporous systems.

Real nanoporous systems employed in intrusion–extrusion applications are inherently complex. Pure-silica zeolites exhibit a wide variety of pore geometries and topologies, whereas functionalized mesoporous silicas with straight channels, such as MCM-41 and SBA-15, contain grafted organic species of different sizes and densities, creating additional structural and chemical heterogeneities that strongly influence wetting thermodynamics. In these materials, pore diameter, interpore distance, wall thickness, and surface chemistry can be independently tuned through synthesis and postsynthetic functionalization.^30–34^ Therefore, we intentionally employed simplified model nanopores designed to isolate the effects of confinement topology and water–surface interactions.

Carbon nanotubes were used as model nanopores with tunable hydrophobicity controlled by varying the Lennard–Jones interaction parameter between water and carbon atoms (ε = 0.06 and 0.07 kcal mol^–1^). These values are lower than those commonly employed for realistic graphite–water systems (ε ≈ 0.09–0.12 kcal mol^–1^).^35,36^ Such reduced interaction strengths were chosen because CNTs described using conventional parameters are spontaneously wetted under ambient conditions, precluding the observation of pressure-induced intrusion–extrusion transitions. They generate effectively hydrophobic nanopores in which water intrusion requires externally applied pressure while reproducing intrusion pressures within the range observed experimentally for hydrophobic nanoporous materials.^37–40^ This approach enables a systematic investigation of the fundamental physical mechanisms governing intrusion and extrusion while minimizing the influence of surface chemistry.

Simulations were performed for a (9,9) single-walled carbon nanotube (SWCNT) with a diameter of approximately 12 Å and a length of 52 Å, corresponding to nanoscale confinement capable of stabilizing quasi-one-dimensional water columns (see Supporting Information (SI) section S1). To examine the combined effects of confinement topology and the presence of an external shell, three simplified model systems were considered: a nanotube open at both ends (CNT), a nanotube surrounded by a hydrophobic shell and open at both ends (sCNT2), and a nanotube surrounded by the same shell and closed at one end (sCNT) (Figures 1a and S1). The hydrophobic shell was inspired by experimentally and computationally studied CNT–Si_3_N_4_ hybrid architectures and serves as a simplified representation of the external environment surrounding nanopores.^41,42^ Details of the model construction and interaction parameters are provided in SI sections S1–S3.

**Figure 1:**
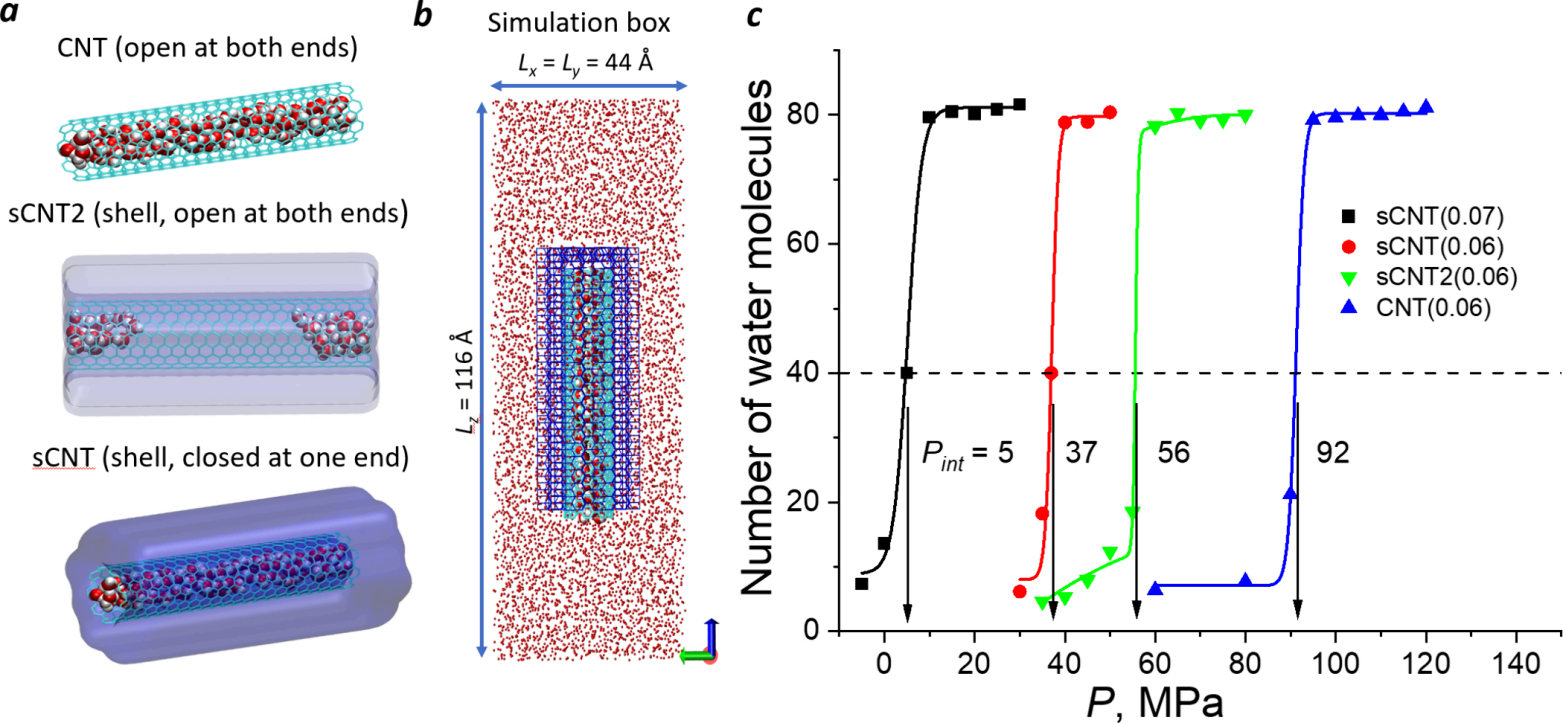
(a) Structural models of CNT, sCNT2, and sCNT nanopores. (b) Simulation box used in the molecular dynamics calculations. Periodic boundary conditions were applied in all three directions. (c) Intrusion isotherms showing the number of confined water molecules as a function of external pressure.

These models, CNT(0.06), sCNT2(0.06), sCNT(0.06), and sCNT(0.07), where the numbers in parentheses denote the value of ε, intentionally neglect many features of realistic porous materials, such as surface heterogeneity, framework flexibility, and the presence of grafted organic species. Nevertheless, they provide a controlled framework for exploring how confinement topology, external interactions, and hydrophobicity jointly determine the thermodynamics and dynamics of confined water.

The nanotubes were immersed in bulk water (Figure 1b), and molecular dynamics simulations were performed in the isothermal–isobaric (*NPT*) ensemble using the DL_POLY package (version 5.1.0).^43^ The simulation protocol follows our previous work.^5,24,25^ Full methodological details, including force-field parameters and representative input files, are provided in the Supporting Information.

Analysis of the full intrusion–extrusion cycles shows that, for all systems, the corresponding isotherms nearly coincide, indicating reversible intrusion/extrusion with negligible hysteresis and molecular spring behavior.^44^ The intrusion pressure, *P*_int_, is defined as the pressure at which the pore is half-filled with water.

The calculated isotherms (Figure 1c) demonstrate that *P*_int_ depends on both the water–carbon interaction strength and the confinement geometry. The sequence CNT → sCNT2 → sCNT separates the contributions of the external shell and confinement topology. For the same value of ε, introducing the outer shell decreases *P*_int_ by 36 MPa, while closing one end of the shell-covered nanotube produces an additional decrease of 19 MPa. Comparison of sCNT(0.06) and sCNT(0.07) isolates the effect of the water–surface interaction strength. Decreasing ε from 0.07 to 0.06 kcal mol^–1^ increases the intrusion pressure of the closed-end system by 32 MPa, reflecting the increased effective hydrophobicity of the nanopore.

## Role of the Shell

The influence of the external shell is consistent with the concept of wall transparency introduced by Kondrat et al.,^45^ who demonstrated that intermolecular interactions across atomically thin confining walls can significantly modify wetting and pore-filling behavior. In bare CNTs, confined water molecules interact across the nanotube wall with the surrounding liquid, including both van der Waals and long-range electrostatic interactions. Introducing the outer shell replaces this external liquid environment with shell atoms, thereby modifying the interactions experienced by confined water molecules. As shown by the interaction energy analysis (SI section S2), water–shell interactions are substantially stronger than the corresponding interactions with the surrounding liquid. Consequently, the shell increases the effective attraction between confined water and its environment, reducing the apparent hydrophobicity of the nanopore and lowering the intrusion pressure.

## Role of Topology and Symmetry

The CNT, sCNT2, and sCNT systems differ in both the interaction environment surrounding the nanotube and confinement topology. As discussed above, the comparison between CNT and sCNT2 isolates the effect of the external shell, whereas comparison between sCNT2 and sCNT isolates the effect of closing one end of the nanotube. As shown below, this topological asymmetry qualitatively changes the intrusion pathway and ultimately affects the thermodynamics of intrusion and extrusion.

Water intrusion into hydrophobic nanopores differs fundamentally from adsorption or capillary condensation. Upon entering a hydrophobic nanopore, the advancing water column forms an interface with the remaining void, analogous to a liquid–vapor interface in macroscopic systems. In CNTs with two open ends, water intrudes from both sides, generating two interfaces that interact as they approach each other inside the pore. In contrast, in sCNT a single interface propagates along the pore and ultimately interacts with the solid wall closing the nanotube. Consequently, the dominant interactions differ: interface–interface interactions in CNT and sCNT2 and interface–wall interactions in sCNT.

These distinctions are particularly important for water, whose dipolar structure is highly sensitive to the balance between water–water, water–wall, and water–void interactions. Confinement induces molecular layering, modifies the hydrogen-bond network, and promotes collective molecular reorientations, resulting in pronounced orientational correlations that directly affect intrusion and extrusion behavior.^5^ Strong confinement can also stabilize ordered water phases.

In carbon nanotubes, a variety of ice nanotube structures have been reported, with the corresponding phases strongly dependent on pore diameter.^20,46^ Phase diagrams for such confined ice structures have been established theoretically,^20,47^ and subsequently confirmed experimentally.^48–50^ These findings demonstrate the strong coupling between confinement geometry, hydrogen-bond topology, and the structure of confined water, suggesting that modifying the intrusion pathway by changing the pore topology may also influence the molecular organization of confined water.

## Structure and Symmetry of Confined Ice

To analyze the structure of confined water, configurations of sCNT(0.06) at *P* = 50 MPa and CNT(0.06) at *P* = 110 MPa were quenched from 300 to 250 K. Under these conditions, water crystallizes inside the nanotube while remaining liquid in the surrounding bulk phase. Depending on pressure and confinement topology, crystallization occurs on time scales ranging from several hundred picoseconds to more than 1 ns. Configurations were recorded every 1 ps to resolve the crystallization process.

Movie S1 reveals that, before crystallization, the confined liquid already exhibits extended regions of correlated molecular orientations together with fragments of locally ordered, ice-like structures (Figure 2a,b). Similar local ordering has previously been reported for water confined in nanotubes with different diameters, cross-sectional geometries, and degrees of hydrophobicity.^5^ Thus, both structural and orientational ordering begin already in the liquid state, well before the formation of the crystalline phase.

**Figure 2:**
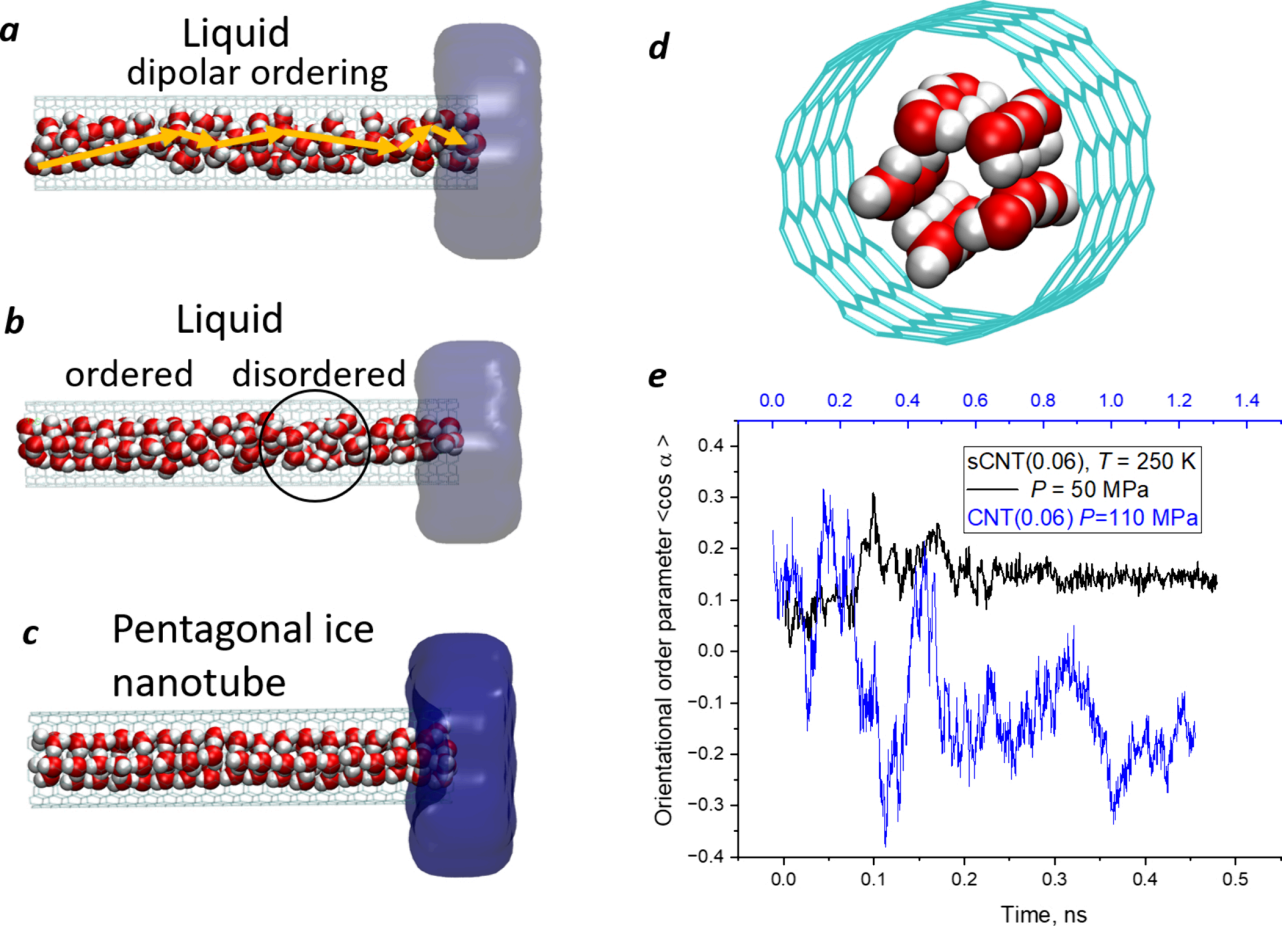
(a) Orientationally correlated domains in confined liquid water. A fragment of the surrounding shell (blue) indicates the closed end of the nanotube. (b) Coexistence of orientationally ordered and disordered regions before crystallization. (c) Pentagonal ice nanotube formed in sCNT(0.06) after quenching to 250 K at *P* = 50 MPa. (d) Cross-sectional view of several stacked pentagonal rings. (e) Evolution of the orientational order parameter, ⟨cos α⟩, during crystallization in two independent simulations.

The crystalline phase formed after quenching consists of stacked pentagonal rings of oxygen atoms with fivefold symmetry along the nanotube axis (Figure 2c,d). The formation of such pentagonal ice nanotubes has been reported previously for carbon nanotubes.^20,46–48^ Our simulations demonstrate that the same pentagonal structure is stabilized in more hydrophobic systems under elevated pressure, indicating that neither the external shell nor the increased hydrophobicity alters the equilibrium crystal structure of confined water. Within each pentagonal ring, three molecular dipoles are oriented approximately parallel to the nanotube axis, whereas two are oriented in the opposite direction, resulting in incomplete dipole compensation.

The evolution of the orientational order parameter, ⟨cos α⟩, is shown in Figure 2e. Here, α is the angle between the molecular dipole moment of an individual water molecule and the nanotube axis. The orientational order parameter is defined as the average value of cos α over all water molecules confined inside the nanotube and therefore characterizes the average molecular alignment with respect to the tube axis. In the following section, we also analyze the orientational sum, ∑_*i*=1_^*N*^ cos α_*i*_, which is proportional to the instantaneous dipole moment of the confined water column.

During crystallization, independent CNT trajectories evolve toward either positive or negative values of ⟨cos α⟩. Although the sign differs between trajectories, the final absolute value is nearly identical, reflecting spontaneous symmetry breaking between two energetically equivalent orientational states. In contrast, the sCNT system consistently evolves toward the same orientation, reflecting the broken axial symmetry introduced by the closed end of the nanotube. As shown below, this asymmetry already induces a finite orientational order in the liquid state before crystallization.

## Dipolar Ordering

The orientationally correlated domains observed in Figure 2 demonstrate that liquid water confined inside the nanotube is not orientationally disordered. To understand the origin of this ordering, we analyze the dipolar organization of the confined water column. The orientational order parameter introduced above provides a quantitative measure of dipole alignment in confined water. Although water remains liquid at 300 K, the orientationally correlated domains visible in Figure 2a,b persist throughout the intrusion–extrusion process. Chains of molecules with correlated dipole orientations extend along the nanotube axis, demonstrating long-range orientational correlations even in the absence of translational order.

These correlations persist during intrusion–extrusion dynamics. Figure 3a,b shows the coupled evolution of the number of confined water molecules and the orientational sum, ∑_*i*=1_^*N*^ cos α_*i*_, which is proportional to the instantaneous dipole moment of the confined water column. In sCNT, the orientational fluctuations are biased toward positive values, indicating persistent net polarization. In contrast, both CNT and sCNT2 exhibit orientational fluctuations centered around zero (Figure 3d), demonstrating the absence of a preferred dipole orientation despite pronounced orientational correlations.

**Figure 3:**
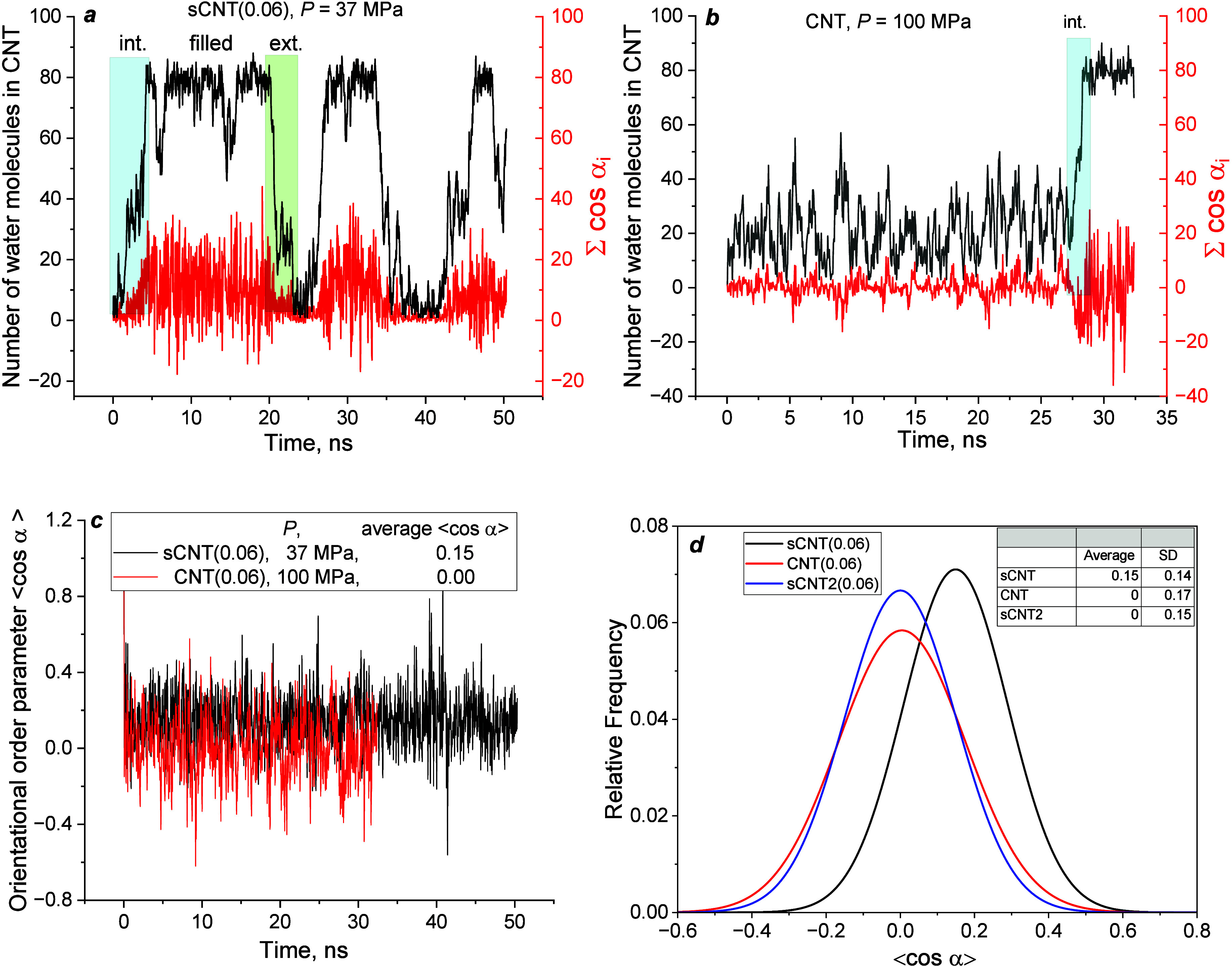
Time evolution of the number of confined water molecules, *N*(*t*) (black), and the orientational sum (red) for (a) sCNT(0.06) and (b) CNT(0.06). Shaded regions mark representative intrusion (blue) and extrusion (green) stages. (c) Time evolution of ⟨cos α⟩. (d) Probability distributions of ⟨cos α⟩ for CNT(0.06), sCNT2(0.06), and sCNT(0.06). Solid lines are Gaussian fits; the inset gives the corresponding mean values and standard deviations.

The comparison between CNT and sCNT2 shows that the external shell alone does not induce a net orientational order. Instead, the finite average orientational order observed in sCNT originates from the broken axial symmetry introduced by closing one end of the nanotube. The probability distributions shown in Figure 3d yield average orientational order parameters of approximately 0.15 for sCNT and nearly zero for both CNT and sCNT2.

The existence of a finite orientational order naturally raises the question of its microscopic origin. Because the orientational correlations extend over the entire confined water column, they cannot be understood solely in terms of individual molecular dipoles. Instead, the collective hydrogen-bond network must be considered. The observed orientational ordering can be rationalized using a simple Ising-like description of hydrogen-bonded chains.^51,52^ Because of the pentagonal arrangement of the chains, not all dipole–dipole interactions can be simultaneously satisfied, resulting in intrinsic geometric frustration.

Each hydrogen-bonded chain is represented by a binary variable *S*_*i*_ = ±1, corresponding to its net dipole orientation. Although dipoles are aligned within individual hydrogen-bonded chains, interchain dipolar interactions favor antiparallel ordering, resulting in an effectively antiferroelectric coupling between neighboring chains.^53^ The interchain interactions can therefore be described by the Hamiltonian

1 $$E=J\underset{\left\langle ij\right\rangle }{\sum }S_{i}S_{j},,J>0$$

where the odd number of chains produces intrinsic geometric frustration (see SI section S4). The model is intended only to illustrate the origin of the orientational degeneracy rather than to provide a quantitative description. As a result, not all dipole–dipole interactions can be simultaneously satisfied, and the lowest-energy states retain a finite residual polarization.

The polarized domains generate bound charges at water–void interfaces and internal electric fields within the pore. As oppositely polarized domains approach each other, the electrostatic interaction between them is expected to increase. An order-of-magnitude estimate (SI section S5) indicates that this contribution is on the order of *k*_B_ *T*, suggesting that electrostatic interactions may contribute to the free energy landscape governing intrusion and extrusion.

Recent experimental and theoretical studies have demonstrated giant and highly anisotropic dielectric responses of strongly confined water, highlighting the central role of collective dipolar ordering under nanoscale confinement.^53–57^ In slit-like geometries, dipolar fluctuations dominate the dielectric response, whereas quasi-one-dimensional confinement promotes collective dipole alignment and the formation of polarized domains. The electrostatic contribution discussed above originates from the collective organization of the hydrogen-bond network. To examine how confinement topology modifies the energetic state of confined water itself, we next analyze the water–water interaction energy.

## Energetics of Confined Water

To determine whether the observed differences in dipolar ordering are accompanied by changes in the energetic state of confined water, we analyzed the average water–water interaction energy of the confined water column as a function of the number of confined water molecules (Figure 4). The analysis includes only pairwise interactions between water molecules simultaneously located inside the nanotube. Interactions with the nanotube wall, the outer shell, and the surrounding bulk water are intentionally excluded to isolate the intrinsic energetics of confined water.

**Figure 4:**
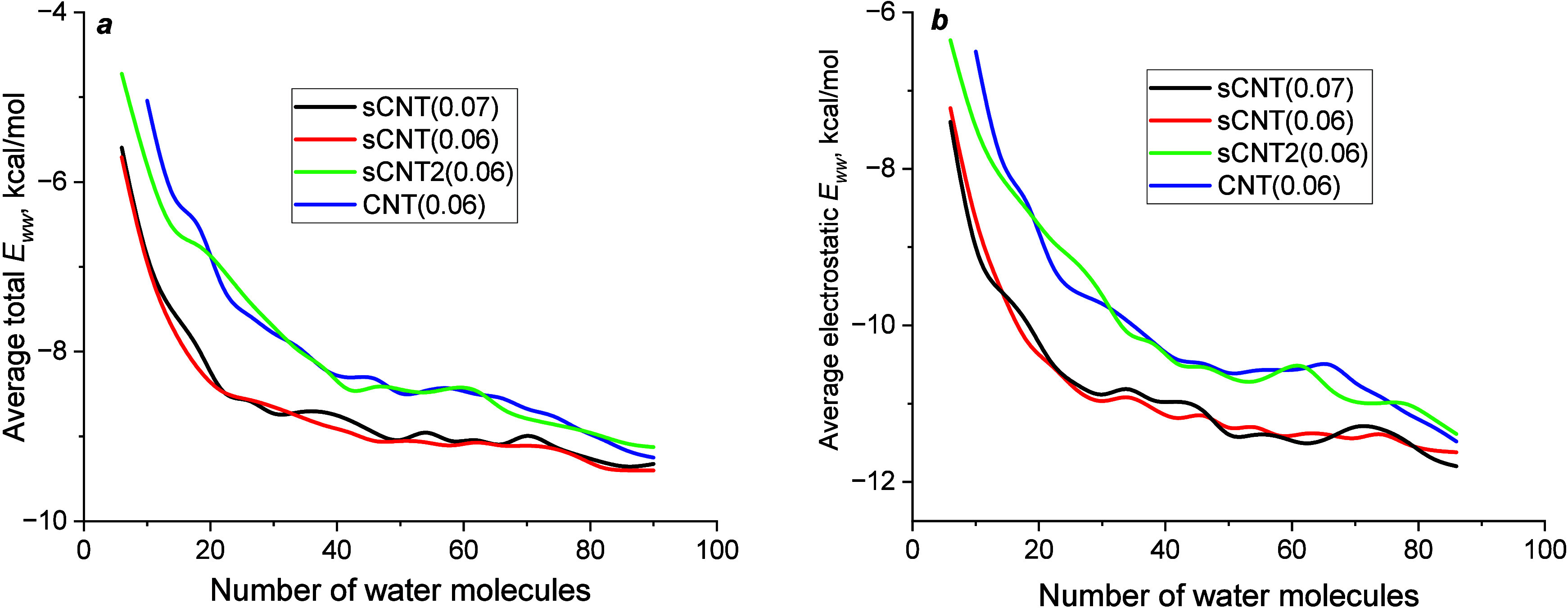
Average interaction energy per confined water molecule as a function of the number of water molecules inside the nanotube. The analysis includes only pairwise interactions between confined water molecules. (a) Total water–water interaction energy. (b) Electrostatic component of the water–water interaction energy.

The total interaction energy (Figure 4a) becomes progressively more negative as the pore fills, reflecting the formation of an increasingly connected hydrogen-bond network. All four systems exhibit nearly identical trends, indicating that confinement topology does not qualitatively alter the local structure of liquid water during intrusion. The electrostatic contribution (Figure 4b) follows the same behavior and accounts for most of the observed energetic differences.

Although the overall water–water interaction energies differ only slightly, a systematic trend is observed. The systems with two open ends (CNT and sCNT2) exhibit consistently less favorable water–water interaction energies than sCNT. This difference correlates with the orientational organization discussed above: in CNT and sCNT2, two energetically equivalent polarization states coexist, leading to the formation of oppositely oriented domains, whereas in sCNT the broken axial symmetry stabilizes a single polarized state. The resulting collective dipolar organization is accompanied by a more favorable energetic state of the confined water column. An independent order-of-magnitude estimate of the electrostatic interaction between polarized domains (SI section S5) yields an energy scale that is comparable to the electrostatic water–water interaction energies obtained here, further supporting the proposed electrostatic contribution to the free energy landscape discussed below.

## Free Energy Landscape and Intrusion–Extrusion Mechanism

To describe the thermodynamics of water intrusion, we consider the number of molecules inside the pore, *N*, as an order parameter. Since the pore exchanges molecules with the surrounding reservoir, the appropriate thermodynamic potential is the grand potential:^2,58,59^

2 $$\Omega \left(N\right)=F\left(N\right)-\mu_{\mathrm{bulk}}N$$

where *F*(*N*) is the constrained Helmholtz free energy (see SI section S6).

Within the approximation of weak compressibility (μ_bulk_ ≈ μ_0_ + *vP*), the corresponding free energy profile can be reconstructed from the probability distribution of *N* as

3 $$G\left(N\right)=-k_{B}T\ln Pr\left(N\right)=\Omega \left(N\right)+\mathrm{const}=\left[F\left(N\right)-\mu_{0}N\right]-PvN+\mathrm{const}$$

which is equivalent to the grand potential up to an additive constant.

Representative time series *N*(*t*) recorded at pressures close to *P*_int_ are shown in Figure 5a. The corresponding probability distributions were used to reconstruct the free energy profiles shown in Figure 5b. The reconstructed free energy profiles reveal systematic differences between the investigated systems. The profiles suggest a progressive decrease in the estimated activation barrier from CNT to sCNT2 and further to sCNT. This trend closely follows the reduction in effective hydrophobicity and the corresponding decrease in the intrusion pressure (Figure 1c). A representative trajectory for sCNT2 at *P* = 55 MPa is provided in Movie S2, where water repeatedly approaches the nanotube entrance without completing intrusion, illustrating the persistence of a finite free energy barrier below *P*_int_. The intermediate region of the CNT profile corresponds to configurations containing two water columns separated by a vapor gap, whereas the sCNT systems contain only a single advancing water column.

**Figure 5:**
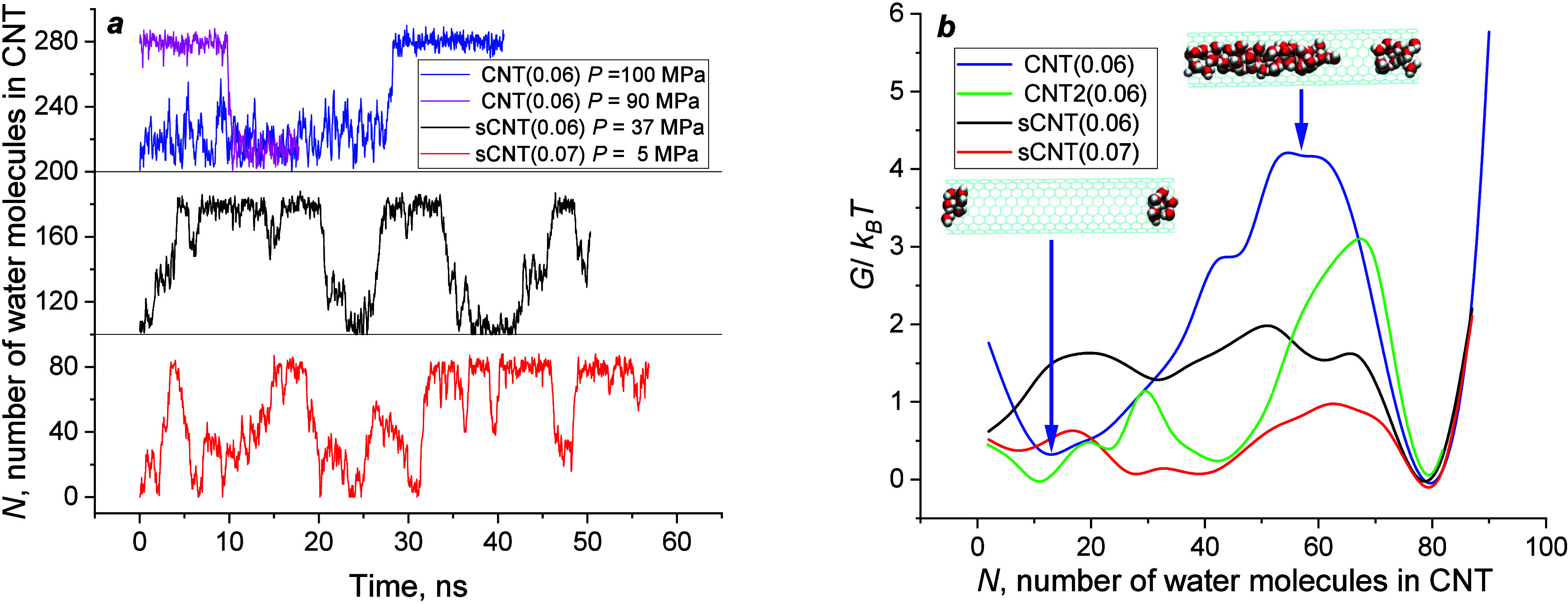
(a) Time series of the number of water molecules inside the nanotube, *N*(*t*), obtained at pressures close to *P*_int_ for CNT and sCNT systems. Curves are offset vertically by 100 water molecules for clarity. (b) Free energy profiles, *G*(*N*), reconstructed from the probability distributions *Pr*(*N*). Insets show representative partially filled configurations.

Because the free energy profiles are reconstructed from finite trajectories, the absolute barrier heights should be regarded as approximate estimates. The discussion, therefore, focuses on robust qualitative features of the free energy landscape, namely the relative stability of the partially filled states and the systematic reduction of the estimated barrier from CNT to sCNT2 and sCNT. Additional consistency tests presented in SI section S7 indicate that these trends are robust despite the limited sampling of rare intrusion–extrusion events.

## Conclusions

We demonstrate that the thermodynamics of water intrusion in hydrophobic nanotubes is governed not only by surface hydrophobicity but also by confinement topology. Although the present study considers simplified model systems, they provide a useful framework for rationalizing experimental observations at the molecular level. As shown previously, the apparent hydrophobicity of nanoporous materials can be tuned by modifying pore architecture, for example, by closing adjacent secondary pores^23,24^ or introducing additional porosity.^25,60^ Such effects cannot be fully described by macroscopic capillary theories such as the Laplace–Washburn or Tolman equations.^15^ Under strong confinement, the intrusion pressure depends not only on pore radius and solid–liquid interactions but also on pore topology, connectivity, geometry, and other structural characteristics of the pore network.^29,44,61^

In the present work, we identify an additional microscopic mechanism governing the apparent hydrophobicity of nanopores. The comparison of CNT, sCNT2, and sCNT systems separates the effects of the external shell and confinement topology. The hydrophobic shell modifies the interaction environment surrounding confined water and reduces the effective hydrophobicity of the nanopore, whereas closing one end of the nanotube breaks axial symmetry and stabilizes a single polarized water domain. As a result, the estimated free energy barrier decreases systematically from CNT to sCNT2 and further to sCNT, following the reduction in intrusion pressure and effective hydrophobicity. These results establish confinement topology as an independent parameter controlling the free energy landscape of confined water.

Our simulations show that orientational ordering develops already in the liquid state prior to crystallization and persists throughout the intrusion process. The resulting collective dipolar organization provides an additional electrostatic contribution to the free energy landscape and establishes a direct connection between the molecular organization of confined water and the thermodynamics of intrusion. More generally, these results suggest that the thermodynamic behavior of nanoconfined water cannot be understood solely in terms of classical capillary theory but must also account for the collective properties of the confined liquid. The identified mechanism of topology-controlled regulation of apparent hydrophobicity opens new opportunities for the rational design of nanoporous materials with tailored wetting properties.

## Supplementary Material









## Notes

Simulation details, additional free energy profiles, structural analyses, orientational distributions, and supplementary figures (PDF)
Movie S1: Crystallization of confined water in sCNT during quenching to 250 K (MPG)
Movie S2: Representative trajectory showing unsuccessful water intrusion into sCNT2 at 55 MPa (MPG)
DL_POLY input files for CNT, sCNT2, and sCNT systems immersed in water (ZIP)
