# Supplementary material for: Topology-Controlled Dipolar Ordering and Wetting Thermodynamics of Water in Hydrophobic Nanotubes
Source: J Phys Chem Lett. 2026 Jul 22;17(31):9016–24. doi: 10.1021/acs.jpclett.6c01820 (PMC13455109; doi:10.1021/acs.jpclett.6c01820)
Supplement: Supplementary file 1 [file jz6c01820_si_001.pdf]

# Supporting Information

# Topology-Controlled Dipolar Ordering and Wetting Thermodynamics of Water in Hydrophobic Nanotubes

*Yuriy G. Bushuev,* Mirosław Chorążewski*

Institute of Chemistry, University of Silesia in Katowice, 40-006 Katowice, Poland

CORRESPONDING AUTHOR INFORMATION

Yuriy G. Bushuev − Institute of Chemistry, University of Silesia in Katowice, 40-006 Katowice, Poland; orcid.org/0000-0001-9463-8627; Email: yuriy.bushuev@us.edu.pl

**S1. Models and System Construction**

Carbon nanotubes were employed as model systems with controlled geometry, topology, and tunable surface interactions. Simulations were performed for a (9,9) single-walled carbon nanotube with a diameter of approximately 12 Å and a length of 52 Å. To investigate how confinement topology and the presence of an external shell influence pressure-induced water intrusion/extrusion, three model systems were considered: a nanotube open at both ends (CNT), a nanotube surrounded by a hydrophobic shell and open at both ends (sCNT2), and a nanotube surrounded by the same shell and closed at one end (sCNT).

The sCNT2 and sCNT models were constructed by surrounding the nanotube with a rigid shell based on the crystallographic structure of β-$Si_3N_4$ (Figure S1a–c). The shell design was inspired by experimentally[1] and computationally[2] studied CNT/$Si_3N_4$ hybrid nanopore architectures, where carbon nanotubes are integrated into silicon nitride membranes for nanofluidic transport. In the sCNT model, the shell additionally closes one end of the nanotube. To simplify the model and minimize the number of adjustable interaction parameters, silicon atoms were replaced by nitrogen atoms occupying the original crystallographic positions of Si atoms. Consequently, the shell should be regarded as a silicon nitride-inspired scaffold rather than a realistic $Si_3N_4$ material. Both the nanotube and the shell were treated as rigid throughout the simulations.

The nanotubes were immersed in bulk water, allowing exchange of molecules between the pore and the surrounding liquid (Figure 1b in the main text). This setup represents an open system in contact with a reservoir and enables the study of pressure-driven wetting and dewetting processes. Representative geometries of the sCNT model are shown in Figure S1d,e. These simplified models enable systematic investigation of how confinement topology, external shell, and water–surface interactions influence intrusion and extrusion.

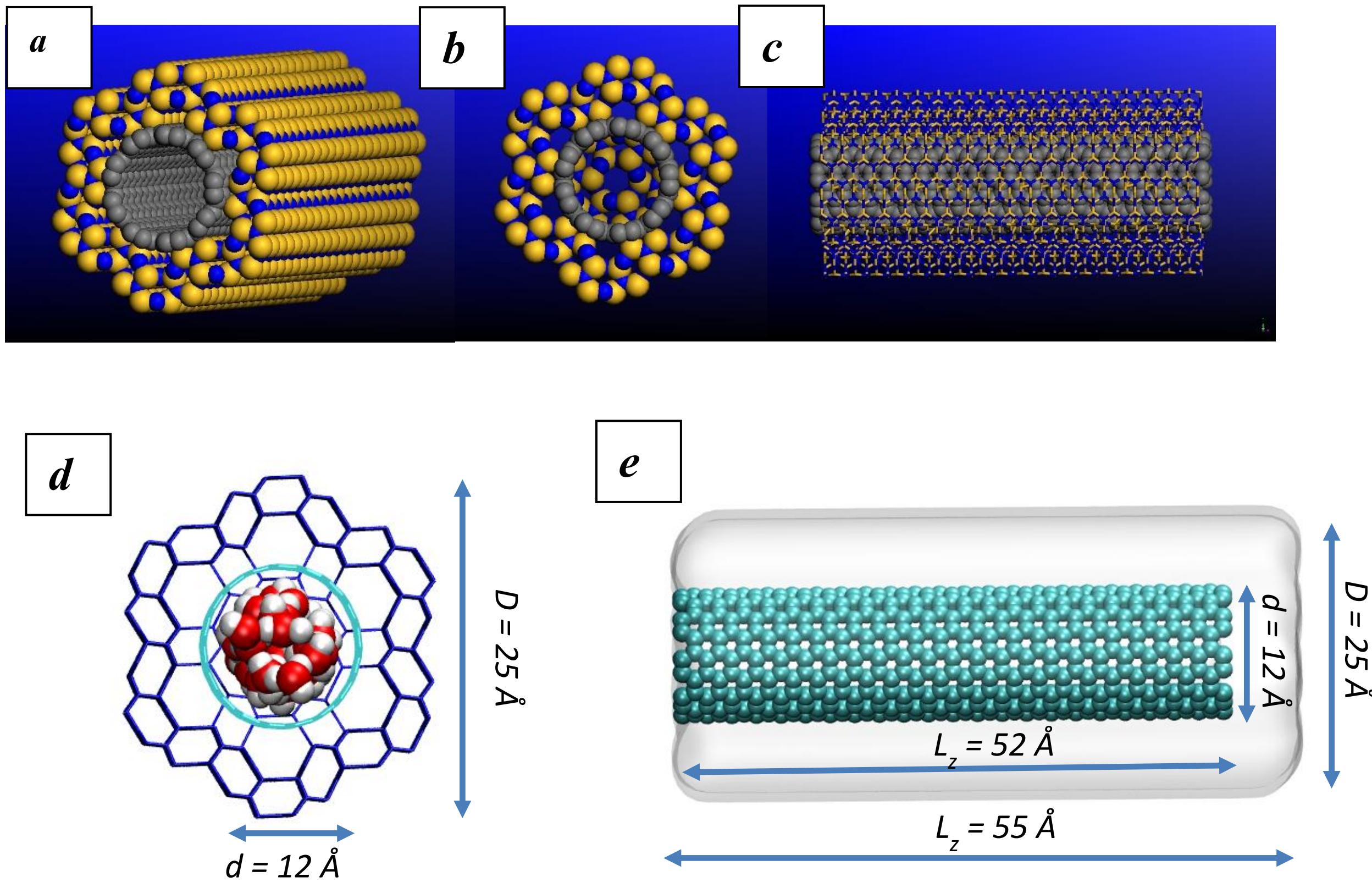


**Figure S1.** Structural models of the nanotube systems. (a) Side view of the (9,9) carbon nanotube surrounded by a rigid shell inspired by the β-$Si_3N_4$ crystal structure (sCNT). (b) View along the tube axis showing that the shell closes one end of the nanotube. (c) Nanotube surrounded by the same shell but open at both ends (sCNT2). Lines indicate the shell framework. (d,e) Geometry of the sCNT model. The shell is shown as atomic connectivity lines (d) or rendered as a transparent body (e) for clarity.

## S2. Interaction Potentials

Interactions between water molecules were described using the flexible SPC/Fw water model.[3] Non-bonded interactions were represented by the Lennard–Jones (LJ) potential in the standard 12–6 form. Interactions between water molecules and carbon atoms of the nanotube were modeled using LJ potentials with a fixed size parameter σ = 3.165 Å and variable well depth ε. To control the effective hydrophobicity of the pore, the ε parameter for C–O interactions was varied and set to 0.06 and 0.07 kcal mol$^{-1}$.

This simplified representation allows independent control of pore topology, external interactions, and effective hydrophobicity, making it possible to isolate the fundamental mechanisms governing intrusion–extrusion processes rather than reproduce a specific material.

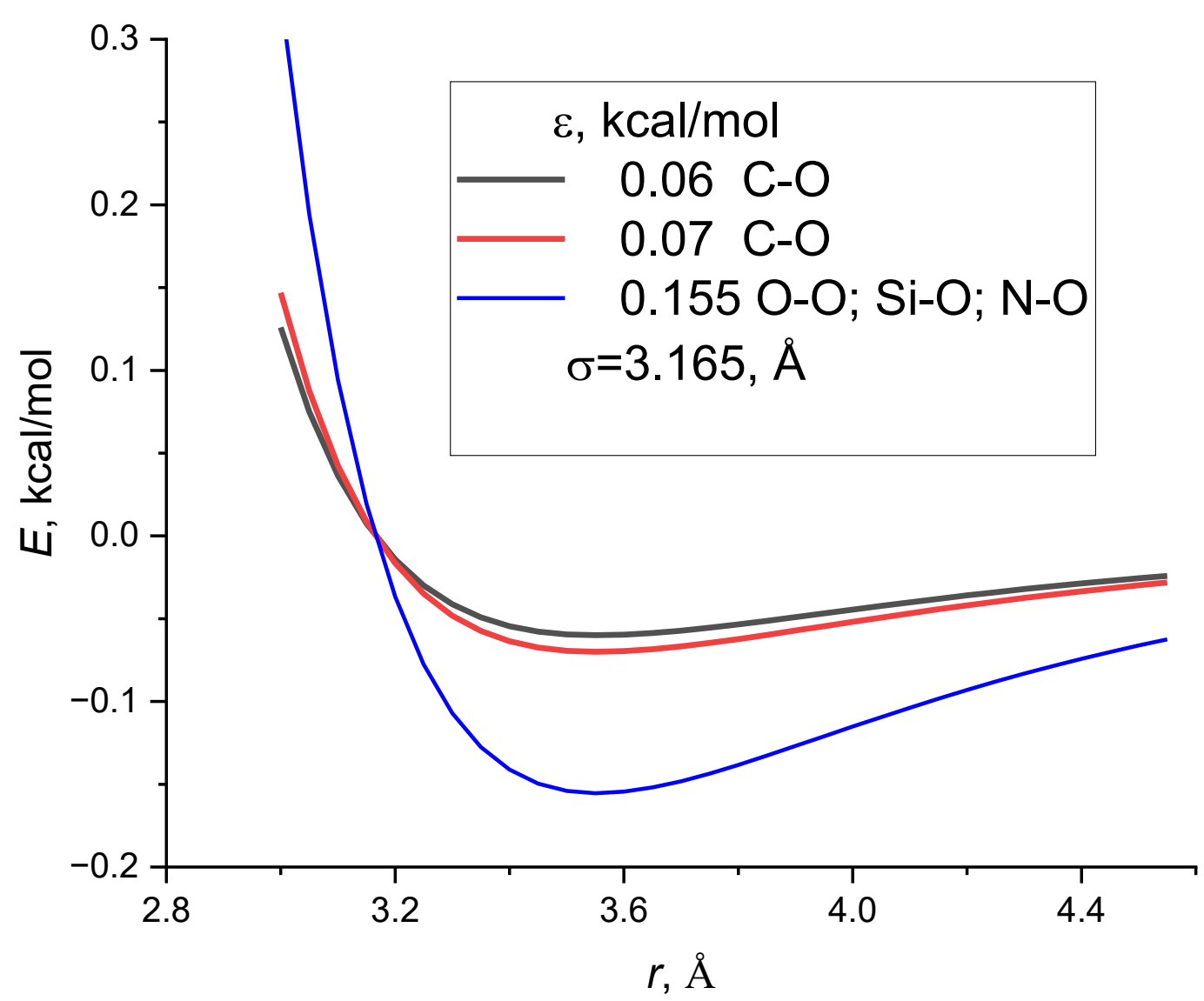


**Figure S2.** Lennard–Jones interaction potentials used in the simulations for water–carbon and water–shell interactions. Different curves correspond to different water–carbon interaction strengths used to control the effective hydrophobicity of the nanopore.

The shell surrounding the nanotube was constructed using a silicon nitride–like structure. For simplicity, both Si and N atoms were represented by identical neutral interaction sites ("N"-type atoms) without partial charges. Interactions between water molecules and shell atoms were described using LJ parameters chosen consistently with those used for water–water interactions, with $\varepsilon = 0.155$ kcal mol$^{-1}$ and $\sigma = 3.165$ Å.

All solid atoms, including carbon atoms of the nanotube and atoms of the shell, were kept fixed during the simulations. Thus, the pore geometry remained unchanged, and only the fluid degrees of freedom were sampled. The interaction potentials used in the simulations are shown in Figure S2 and are provided in the FIELD file of the DL_POLY package.

In the present model, the nanotube walls and the outer shell are represented by rigid nonpolarizable Lennard–Jones interaction sites. No explicit dielectric polarization or electrostatic screening by the solid matrix is included. Consequently, the observed wall-thickness effects do not originate from electrostatic transmission through the solid wall itself.

Instead, the effective hydrophobicity of the nanopore is governed by competition between intermolecular interactions across the pore boundary and direct water–wall interactions. In bare CNT systems, the atomically thin nanotube wall allows confined water molecules to interact with external bulk water across the atomically thin wall, including long-range electrostatic interactions between confined and external water molecules. Such cross-wall interactions are analogous to the wall-transparency effects discussed by Kondrat et al.[4]

When the nanotube is surrounded by a rigid outer shell, the external water is replaced by neutral Lennard–Jones interaction sites. This modification affects the system in two competing ways. First, it suppresses direct interactions between confined water and the external bulk liquid. Second, the shell atoms introduce additional Lennard–Jones interactions with confined water molecules,

increasing the effective water–solid attraction. In the present systems, the latter effect dominates, leading to a reduction of the apparent hydrophobicity and a lower intrusion pressure.

To clarify the microscopic origin of the shell effect, the interaction energy of confined water molecules was decomposed into contributions arising from the CNT wall, the surrounding liquid, and the shell. Only molecules located within the central 30 Å segment of the nanotube were analyzed in order to eliminate end effects. Interaction energies were evaluated inside a cylindrical region with a radius of 13 Å centered on the nanotube axis. In the bare CNT, this region contains surrounding bulk water, whereas in shell-covered systems, it is occupied predominantly by shell atoms.

**Table S1.** Average interaction energies (kcal $mol^{-1}$ per water molecule) between water molecules confined within the central 30 Å segment of the nanotube and their external environment. Interaction energies were evaluated inside a cylindrical region of radius 13 Å centered on the nanotube axis. Only interactions of confined water with the nanotube wall, shell atoms, or surrounding bulk water were included. Interactions among water molecules within the analyzed nanotube segment were excluded.

| **System** | **Total external interaction** | **Water-C** | **Water-Shell** | **Water-Water** |
|---|---|---|---|---|
| sCNT(0.07) | -1.71 | -1.19 | -0.52 | — |
| sCNT(0.06) | -1.70 | -1.18 | -0.52 | — |
| sCNT2(0.06) | -1.37 | -0.97 | -0.40 | — |
| CNT(0.06) | -1.31 | -1.09 | — | -0.22 |

**Abbreviations:** *Water–C* denotes the interaction energy between confined water molecules and carbon atoms of the nanotube; *Water–Shell* denotes the interaction with shell atoms; *Water–Water* denotes the interaction with surrounding bulk water located outside the nanotube but within the analysis cylinder.

The calculated energies presented in Table S1 demonstrate that the shell substantially modifies the interaction environment experienced by confined water molecules. In the bare CNT, the

interaction with the surrounding liquid amounts to only −0.22 kcal $mol^{-1}$ per water molecule, whereas the shell contributes considerably stronger attractive interactions (−0.52 kcal $mol^{-1}$ in sCNT and −0.40 kcal $mol^{-1}$ in sCNT2). Consequently, replacing the surrounding water with shell atoms increases the total attractive interaction experienced by confined water molecules.

The evolution of the interaction energies closely follows the experimentally observed sequence of intrusion pressures, CNT (92 MPa) > sCNT2 (56 MPa) > sCNT (37 MPa). This correlation indicates that the reduction in intrusion pressure is primarily due to changes in the external interaction environment rather than to changes in the nanotube itself. The shell, therefore, reduces the apparent hydrophobicity of the nanopore by replacing weak interactions with surrounding water with stronger direct interactions with shell atoms.

Interestingly, decreasing the water–carbon interaction parameter from $\varepsilon = 0.07$ to 0.06 kcal $mol^{-1}$ produces almost no change in the average interaction energy of the fully intruded state, despite increasing the intrusion pressure by approximately 32 MPa. This result reflects the fact that the present analysis considers only the completely filled nanotube. The intrusion pressure is determined by the free-energy difference between dry and filled states, rather than by the potential energy of the filled configuration alone.

The topology-induced effects discussed in the main text originate from a different mechanism associated with symmetry breaking in nanotubes closed at one end and should therefore be regarded as complementary to the wall-transparency effect rather than as its direct consequence.

## S3. Molecular Dynamics Simulations

The nanotubes were immersed in orthorhombic simulation boxes containing 6800 water molecules. Periodic boundary conditions were applied in all directions to eliminate surface effects. Molecular dynamics simulations were performed in the NPT ensemble at $T$ = 300 K and $P$ = 0–150 MPa using the DL_POLY package (version 5.1.0).[5] Temperature and pressure were controlled using the Melchionna modification of the Nosé–Hoover algorithm, with relaxation times of 0.5 ps and 1 ps for the thermostat and barostat, respectively. For simplicity, the nanotubes and the surrounding shell were treated as rigid bodies, with all atoms fixed at their crystallographic positions. This approach ensures structural stability of the pore during the simulations.

Water was modeled using the flexible SPC/Fw model, with a recommended spherical cut-off radius of 9 Å for short-range interactions.[3] The Smooth Particle Mesh Ewald (SPME) method was used to compute long-range electrostatic interactions. The equations of motion were integrated using the velocity Verlet algorithm with a time step of 1 fs. The duration of simulations depended on the applied pressure and system configuration, reaching up to 60 ns in selected cases. Representative input files (CONTROL, FIELD, and CONFIG) containing full simulation details are provided in the Supporting Information.

The chosen simulation protocol follows our previous works.[6–9]

**S4. Dipole–Dipole Interaction Model and Ising Mapping**

The dipolar ordering in confined water can be rationalized in terms of dipole–dipole interactions. The interaction energy between two dipoles μ separated by a distance r scales as $\mu^2/(4\pi\varepsilon_0 r^3)$, with angular dependence determining whether configurations are energetically favorable or unfavorable. Head-to-tail alignment minimizes the interaction energy, whereas parallel side-by-side configurations are energetically unfavorable.

In the pentagonal ice structure formed inside the nanotube, water molecules organize into hydrogen-bonded chains aligned along the tube axis. Within each chain, dipoles are strongly correlated and approximately collinear, allowing each chain to be represented by an effective binary variable $S_i = \pm 1$ corresponding to its net polarization direction.

The total interaction energy between chains can be expressed as a sum of dipole–dipole interactions between molecules belonging to different chains. Due to the $r^{-3}$ decay and geometric separation between chains, nearest-neighbor interactions dominate, leading to an effective low-dimensional description.

The resulting Hamiltonian takes the form of an effective antiferromagnetic Ising model on an odd cycle analogous to geometrically frustrated spin systems:[10,11]

$$E = J \sum_{<ij>} S_i S_j\,, \quad J > 0 \qquad \text{(S1)}$$

where the sum runs over nearest-neighbor chains. This system is intrinsically frustrated: although each interaction energetically favors antiparallel alignment, the cyclic topology prevents simultaneous satisfaction of all pairwise interactions. As a consequence, at least one bond remains frustrated in the ground state.

The resulting minimal-energy manifold corresponds to (3+2) configurations, in which three chains share one polarization direction, and two adopt the opposite orientation. The degeneracy of

this manifold originates from the translational freedom of the frustrated bond along the five-site ring. Thus, the pentagonal ice structure can be viewed as a direct manifestation of geometric frustration arising from dipolar interactions under pentagonal confinement. The energy difference between competing configurations, such as (3+2) and (4+1), is on the order of J per frustrated bond and is therefore comparable to $k_BT$ in the relevant temperature range. This explains the presence of transient (4+1) configurations in the liquid state and the sampling of different members of the degenerate (3+2) manifold during crystallization.

As a result, the confined water system represents a frustrated dipolar assembly exhibiting a residual polarization. This net dipole moment arises from the incomplete cancellation of antiparallel chain contributions imposed by geometric frustration. The observed polarization thus emerges from the interplay between dipole–dipole interactions, hydrogen-bond constraints, and symmetry-imposed cancellation of dipolar order.

## S5. Estimate of Electrostatic Interaction Energy

The net dipole moment of a confined water column can be expressed as

$$M = \Sigma\, \mu \cos \alpha_i = N\, \mu\, \langle \cos \alpha \rangle, \qquad \text{(S2)}$$

where $N$ is the number of molecules in the column, μ is the molecular dipole moment, and $\langle \cos \alpha \rangle$ is the orientational order parameter.

For the present system, the maximum of the free-energy barrier is observed at $N \approx 60$, corresponding to the coexistence of two water domains separated by a void region. Given the total occupancy of approximately 80 molecules in a fully filled nanotube of length ≈ 5.2 nm (52 Å), the characteristic separation between the two domains is about 1.3 nm.

Assuming two approximately symmetric domains containing $N \approx 30$ molecules each, and using $\mu \approx 2.3$ D for the SPC/Fw model and $\langle \cos \alpha \rangle \approx 0.15$, the dipole moment of a single domain can be estimated as

$$M \approx 30 \times 2.3 \times 0.15 \approx 10 \text{ D}. \quad \text{(S3)}$$

The dipole–dipole interaction energy between two oppositely polarized domains separated by a distance $r \approx 1.3$ nm is then given by

$$U \approx (1 / 4\pi\varepsilon_0) \times M^2 / r^3. \quad \text{(S4)}$$

Substituting the above values yields an interaction energy on the order of $U \approx (0.5–1)\ k_BT$. This order-of-magnitude estimate is consistent with the difference in the average electrostatic water–water interaction energies reported in Figure 4b of the main text (approximately 1 kcal $mol^{-1}$, corresponding to about 1.7 $k_BT$ at N=50–60). The latter quantity is expected to be larger because it includes not only the long-range interaction between polarized domains but also local electrostatic rearrangements within the hydrogen-bond network.

Since the polarized domains are separated by a low-density void region inside the nanotube, dielectric screening is expected to be substantially reduced. The present analysis should therefore be regarded as an order-of-magnitude approximation. Nevertheless, it indicates that electrostatic interactions between polarized water domains are comparable to thermal energy and may therefore provide a measurable contribution to the free-energy barrier associated with domain coalescence.

**S6. Free-Energy Formalism**

We consider a nanopore in contact with a bulk liquid reservoir at temperature *T* and pressure *P*. Since the pore exchanges water molecules with the surrounding liquid, the appropriate thermodynamic description is based on the grand canonical ensemble.[12–14]

To characterize pore filling, we introduce an order parameter *N*, defined as the number of water molecules inside the pore. The probability distribution of *N* can be expressed as a projection of the configurational ensemble onto this coordinate:

$$Pr(N) = \frac{1}{\Xi}\sum_i \exp[-\beta(E_i - \mu_{bulk}N_i)]\delta(N - \boldsymbol{\mathcal{N}}(i)), \quad \text{(S5)}$$

where $E_i$ and $N_i$ are the energy and total number of molecules in configuration $i$, $\boldsymbol{N}(i)$ is the number of molecules inside the pore, $\mu_{bulk}$ is the chemical potential of the reservoir, and $\Xi$ is the grand partition function.

The constrained (Landau) Helmholtz free energy associated with the order parameter *N* is defined as:[13,15]

$$e^{-\beta F(N)} \equiv \sum_i \exp(-\beta E_i)\delta(N - \boldsymbol{\mathcal{N}}(i)). \quad \text{(S6)}$$

Substituting this definition into the expression for *Pr*(*N*) yields

$$Pr(N) \propto \exp[-\beta(F(N) - \mu_{bulk}N)], \quad \text{(S7)}$$

which defines the grand potential as a function of the order parameter:

$$\Omega(\mathrm{N}) = F(N) - \mu_{bulk}N. \quad \text{(S8)}$$

Accordingly,

$$Pr(N) \propto \exp[-\beta\Omega(N)], \quad \text{(S9)}$$

and the free-energy profile reconstructed from molecular simulations is given by

$$G(N) = -k_BT \ln Pr(N) = \Omega(N) + \text{const.} \quad \text{(S10)}$$

Thus, the potential of mean force $G(N)$ obtained from simulations corresponds to the grand potential governing pore filling, up to an additive constant.

Although the simulations are performed in the NPT ensemble, this formulation provides an effective thermodynamic description in which the chemical potential of the reservoir is controlled by pressure. The resulting free-energy profile $G(N)$ can therefore be interpreted as the grand potential as a function of pore occupancy.

**S7. Probability Distributions and Free-Energy Reconstruction**

The probability of observing a configuration with exactly $N$ water molecules in the pore, $Pr(N)$, was obtained from the time series $N(t)$ by histogramming the trajectory using bins of width $\Delta N = 4$ and normalizing by the total number of samples. Results were found to be insensitive to moderate variations of bin size.

For the sCNT systems, multiple spontaneous intrusion–extrusion events were observed within a single simulation run, allowing direct estimation of $Pr(N)$ and the corresponding free-energy profile. For CNT systems, such transitions are rare on accessible time scales. To improve sampling, probability distributions were constructed separately for intrusion at $P$ = 100 MPa and extrusion at $P$ = 90 MPa. The final distribution $Pr(N)$ was obtained by averaging these datasets, assuming that both conditions remain sufficiently close to coexistence.

To assess the robustness of the reconstructed free-energy profiles, we performed an additional analysis for the sCNT(0.06) system. The probability distribution $Pr(N)$ obtained at the coexistence pressure ($P$ = 37 MPa) was compared with distributions averaged over simulations at higher

pressures ($P = 40$–$50$ MPa), where only intrusion events were observed. The corresponding results are shown in Figure S3a.

Because only intrusion events contribute at higher pressures, the filled state is overrepresented, while the empty state is underestimated. Nevertheless, the distributions obtained at different pressures exhibit similar shapes and a dominant maximum corresponding to the filled state.

The corresponding free-energy profiles $G(N)$ are shown in Figure S3b. As expected, the profiles reconstructed from higher-pressure simulations exhibit a systematic tilt toward the filled state due to the shift in chemical potential. This effect can be corrected by applying a linear transformation of the form $a + bN$. Within the approximation $\mu \approx \mu_0 + vP$, this transformation corresponds to the pressure-dependent shift in the grand potential.

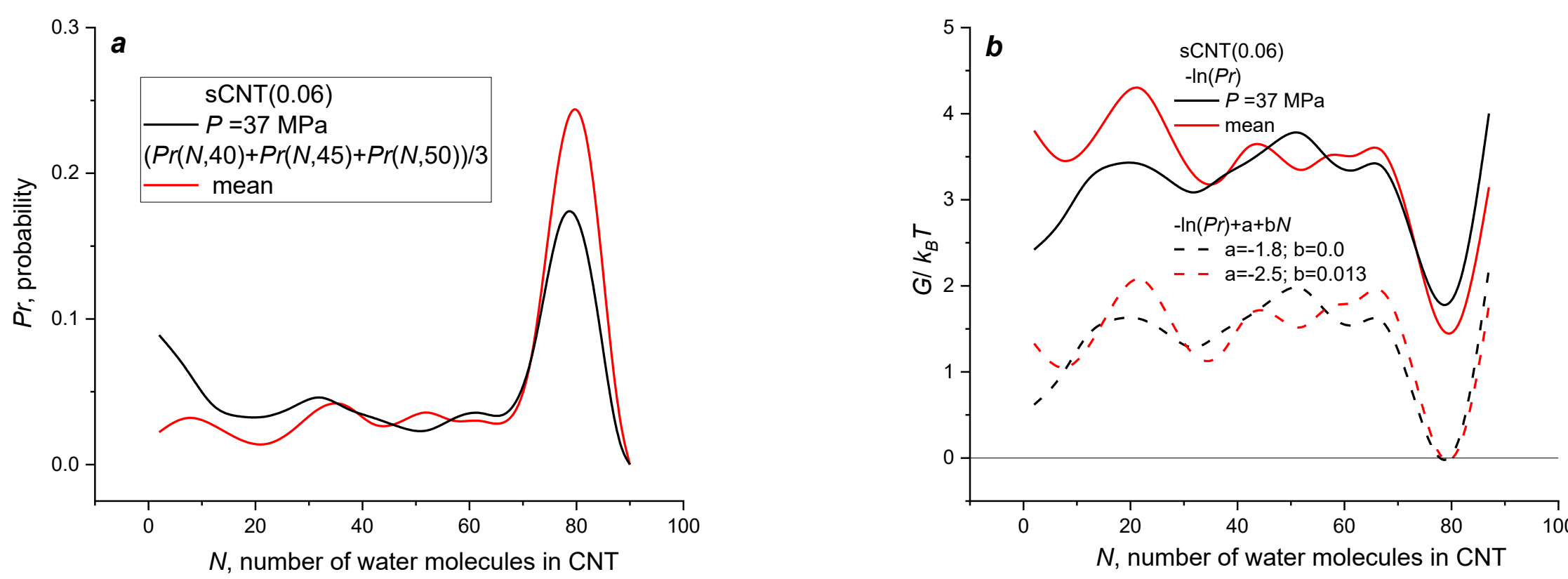


**Figure S3.** (a) Probability distributions $Pr(N)$ for the sCNT(0.06) system. (b) Free-energy profiles $G(N)$. Black curves correspond to $P = 37$ MPa. Red curves correspond to profiles reconstructed from averaged distributions at higher pressures (40–50 MPa, intrusion only). Dashed lines: profiles after linear correction accounting for the pressure difference.

After applying this correction, the free-energy profiles obtained at different pressures show good agreement in the region of overlap. This indicates that the main features of the free-energy landscape, including barrier heights, are relatively insensitive to sampling limitations.

The reconstructed profiles show that the free-energy barrier in sCNT does not exceed ~2 $k_BT$ and that the intermediate region is nearly flat. The free energy associated with the first nonzero occupancy ($N \approx 2$) is on the order of $k_BT$ and can be attributed to the formation of a liquid–vapor interface (meniscus) inside the pore.

---

**Supporting Information References**


(1) Holt, J. K.; Noy, A.; Huser, T.; Eaglesham, D.; Bakajin, O. Fabrication of a Carbon Nanotube-Embedded Silicon Nitride Membrane for Studies of Nanometer-Scale Mass Transport. *Nano Lett.* **2004**, *4* (11), 2245–2250. https://doi.org/10.1021/nl048876h.

(2) Si, W.; Chen, H.; Wu, G. Regulation of Protein Translocation through A Si3N4-CNT Stacked Nanopore Using an Embedded Gold Nanoparticle. *Phys. Chem. Chem. Phys.* **2025**, *27* (27), 14229–14239. https://doi.org/10.1039/d5cp01017a.

(3) Wu, Y.; Tepper, H. L.; Voth, G. A. Flexible Simple Point-Charge Water Model with Improved Liquid-State Properties. *J. Chem. Phys.* **2006**, *124*, 024503. https://doi.org/10.1063/1.2136877.

(4) Kondrat, S.; Schimmele, L.; Giacomello, A.; Tasinkevych, M.; Dietrich, S. Manipulating Wetting and Pore Filling by Wall Transparency. *Commun Phys* **2025**, *8* (328), 1–6. https://doi.org/10.1038/s42005-025-02239-2.

(5) Todorov, I. T.; Smith, W.; Trachenko, K.; Dove, M. T. DL_POLY_3: New Dimensions in Molecular Dynamics Simulations via Massive Parallelism. *J. Mater. Chem.* **2006**, *16* (20),

1911–1918. https://doi.org/10.1039/b517931a.

(6) Bushuev, Y. G.; Grosu, Y.; Chorążewski, M. A.; Meloni, S. Effect of the Topology on Wetting and Drying of Hydrophobic Porous Materials. *ACS Appl. Mater. Interfaces* **2022**, *14* (26), 30067–30079. https://doi.org/10.1021/acsami.2c06039.

(7) Bushuev, Y. G.; Grosu, Y.; Chorążewski, M. A.; Meloni, S. Subnanometer Topological Tuning of the Liquid Intrusion/Extrusion Characteristics of Hydrophobic Micropores. *Nano Lett.* **2022**, *22* (6), 2164–2169. https://doi.org/10.1021/acs.nanolett.1c02140.

(8) Bushuev, Y. G.; Grosu, Y.; Chorążewski, M. Spontaneous Dipole Reorientation in Confined Water and Its Effect on Wetting/Dewetting of Hydrophobic Nanopores. *ACS Appl. Mater. Interfaces* **2024**, *16* (6), 7604–7616. https://doi.org/10.1021/acsami.3c17272.

(9) Bushuev, Y. G. Effects of Size and Porosity on the Hydrophobicity of Hierarchical Nanoparticles. *Nano Lett.* **2025**, *25* (8), 3351–3356. https://doi.org/10.1021/acs.nanolett.5c00058.

(10) *Introduction to Frustrated Magnetism: Materials, Experiments, Theory*; Lacroix, C., Mendels, P., Mila, F., Eds.; Springer: Berlin, Heidelberg, 2011. https://doi.org/10.1007/978-3-642-10589-0.

(11) Moessner, R.; Ramirez, A. P. Geometrical Frustration. *Phys. Today* **2006**, No. February, 24–29. https://doi.org/10.1063/1.2186278.

(12) Alba-Simionesco, C.; Coasne, B.; Dosseh, G.; Dudziak, G.; Gubbins, K. E.; Radhakrishnan, R.; Sliwinska-Bartkowiak, M. Effects of Confinement on Freezing and Melting. *J. Phys. Condens. Matter* **2006**, *18* (6). https://doi.org/10.1088/0953-8984/18/6/R01.

(13) Frenkel, D.; Smit, B. *Understanding Molecular Simulation: From Algorithms to Applications*, 2nd ed.; Academic Press: San Diego, 2002.

(14) Gelb, L. D.; Gubbins, K. E.; Radhakrishnan, R.; Sliwinska-Bartkowiak, M. Phase Separation in Confined Systems. *Reports Prog. Phys.* **1999**, *62* (12), 1573–1659. https://doi.org/10.1088/0034-4885/62/12/201.

(15) Chandler, D. *Introduction to Modern Statistical Mechanics*; Oxford University Press: New York, 1987.

(16) Bocquet, L.; Charlaix, E. Nanofluidics, from Bulk to Interfaces. *Chem. Soc. Rev.* **2010**, *39* (3), 1073–1095. https://doi.org/10.1039/b909366b.
